# Supplementary material for: Nonlinear biomarker interactions in conversion from mild cognitive impairment to Alzheimer's disease
Source: Hum Brain Mapp. 2020 Jul 9;41(15):4406–18. doi: 10.1002/hbm.25133 (PMC7502835; doi:10.1002/hbm.25133)
Supplement: Supplementary file 1 — Appendix S1: Supporting Information [file HBM-41-4406-s001.docx]

# Supplementary material

## Figures

Figure S1. Univariate plots for Model 3: Main effects and two-way interactions between amyloid-β-PET and hippocampal volume.


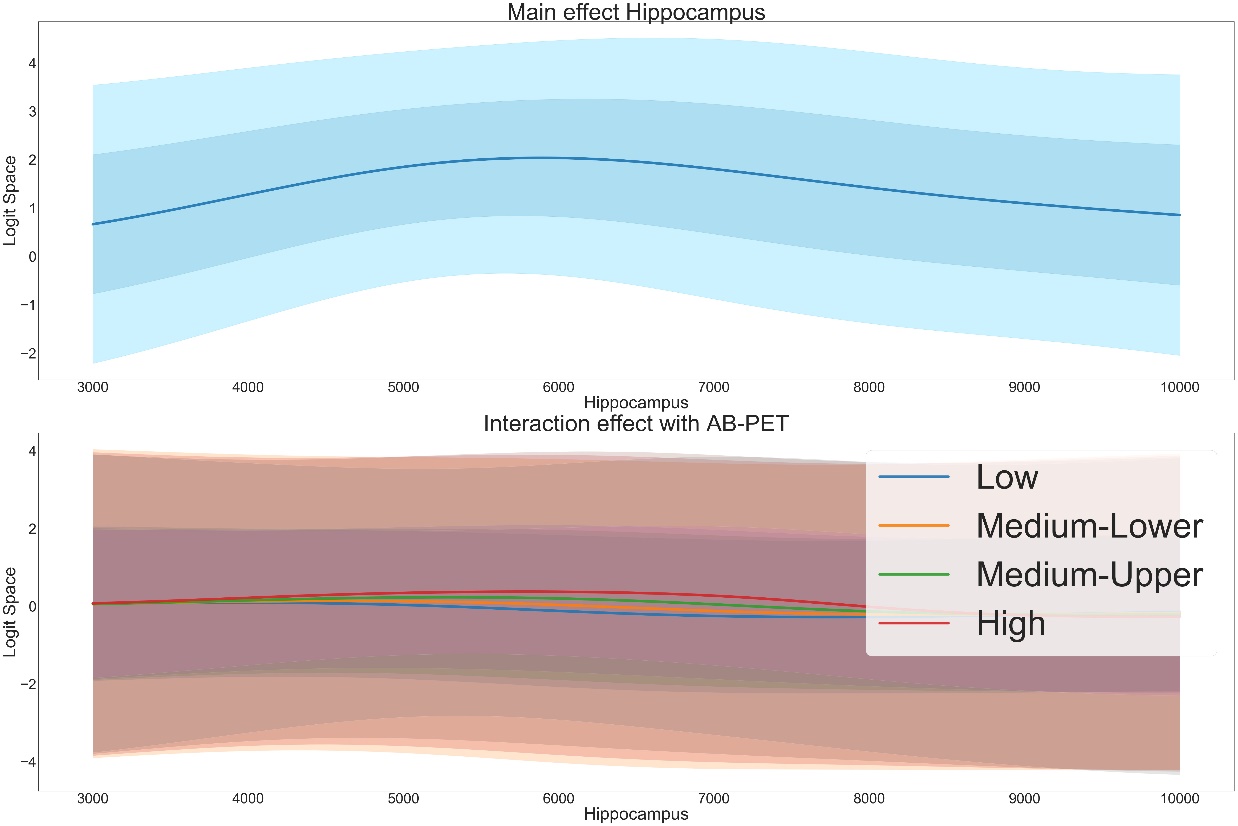


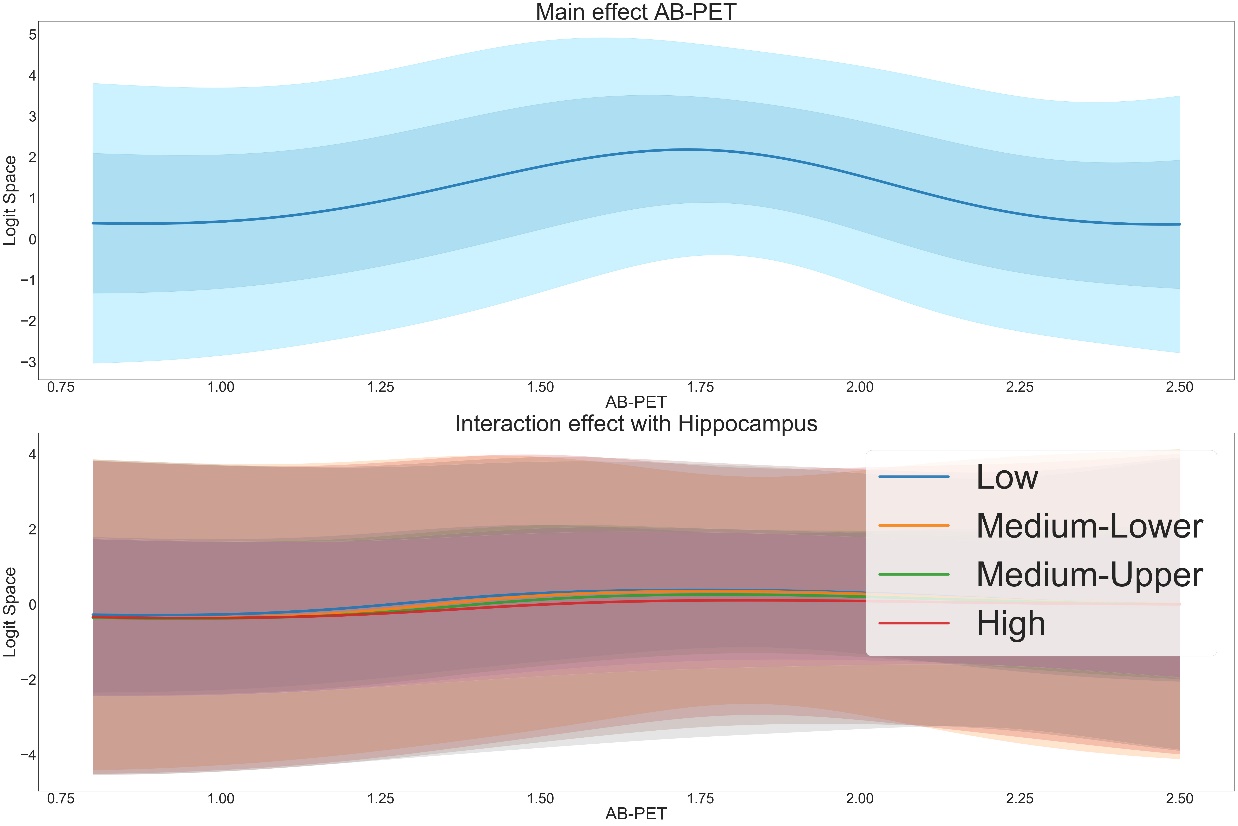


Univariate plots show the stable MCI (lower values) versus progressive MCI (higher values) predictions in logit space. First rows in subfigures illustrate the dynamics of the main effects kernel associated to the biomarker, whereas the second rows in subfigures show the dynamics of the predictions stemming from the bivariate interaction kernel where we take anchor values for the other covariate (low: 20th percentile, medium-lower: 40th percentile, medium-upper: 60th percentile, high: 80th percentile).

Figure S2. Interaction Detection Framework for Additive Gaussian Processes


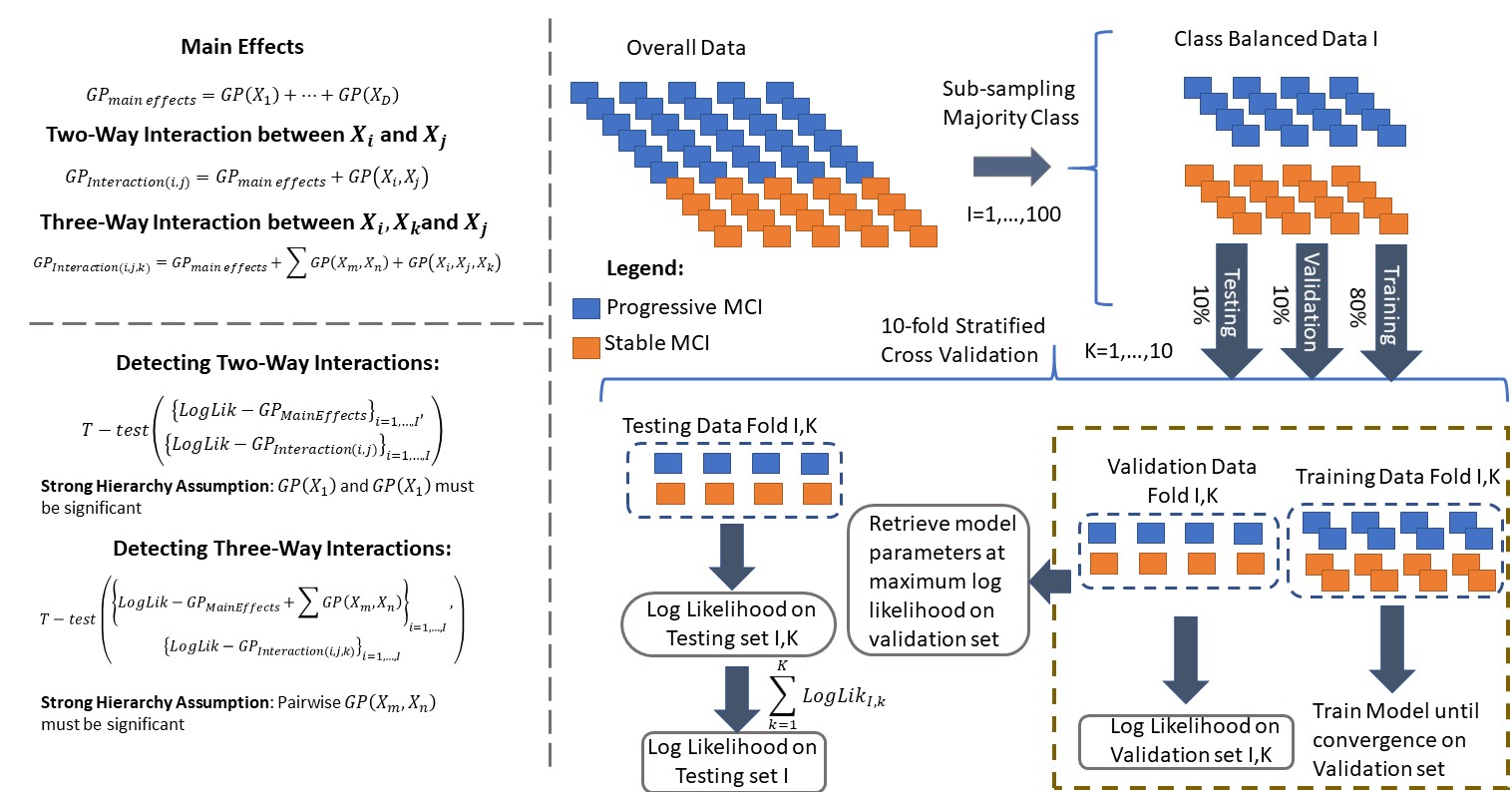


**Right panel:** To address the class imbalance between progressive MCI and stable MCI, we sub-sampled the majority class 100 times, arriving at 100 different class balanced bootstrapped datasets. Within each bootstrapped dataset, we use a 10-fold stratified cross-validation scheme to generate training, validation and test sets. A given Gaussian Processes architecture is trained on the training set until convergence on the validation set. To obtain the log likelihood values for the testing set we retrieve the model parameters for the highest log likelihood value on the validation set. For each bootstrapped dataset, we add up the log likelihood values from each fold. Finally, each model has 100 different bootstrapped testing set log likelihood values.

**Left panel:** To detect two-way interactions a paired t-test is performed between the log likelihood values stemming from a main effects model (each covariate has a separate Gaussian Process with univariate kernels) and a two-way interaction model (main effects model plus two-way interaction kernel between the covariates in question). The same logic is applied for detecting three-way interactions, with the difference that the null hypothesis model consists of the main effects alongside all possible two-way interactions between the three covariates tested for.

Figure S3. Contour plots for Model 1: Two-way interaction between CSF amyloid-β42 and hippocampal volume, stratified by brain-PAD.


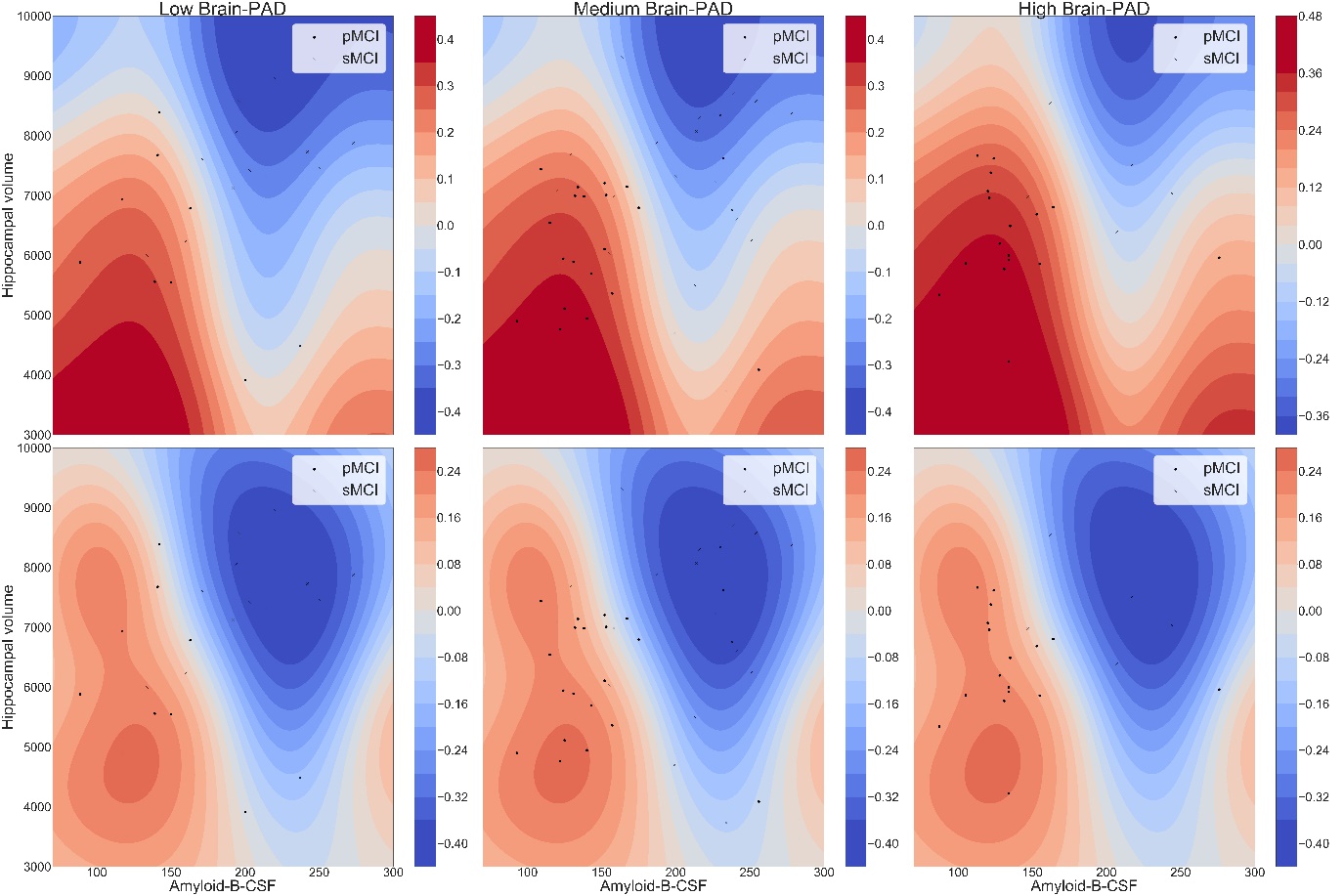


Contour plots show the stable MCI versus progressive MCI decision boundary in biomarker space. First row illustrates the dynamics of the summation of the independent univariate kernels as Brain-PAD is increased from -4.104 (low Brain-PAD) to 0.575 (medium Brain-PAD) and then finally to 4.411 (high Brain-PAD). Second row shows just the effect of the bivariate kernel between CSF amyloid-β42 and hippocampal volume. All plots denote the relative contribution of the respective kernel to the final decision boundary landscape. Points correspond to subjects (stable MCI and progressive MCI) from a class-balanced subset of the overall dataset. Positive values indicate a higher chance of conversion to Alzheimer’s disease within three years. Comparison of the first row (additive model) and second row (interactive model) conveys the influence of incorporating nonlinear interaction kernels on the biomarker space.

Figure S4. Contour plots for Model 2: Two-way and three-way interactions between amyloid-β-PET and hippocampal volume, stratified by brain-PAD.


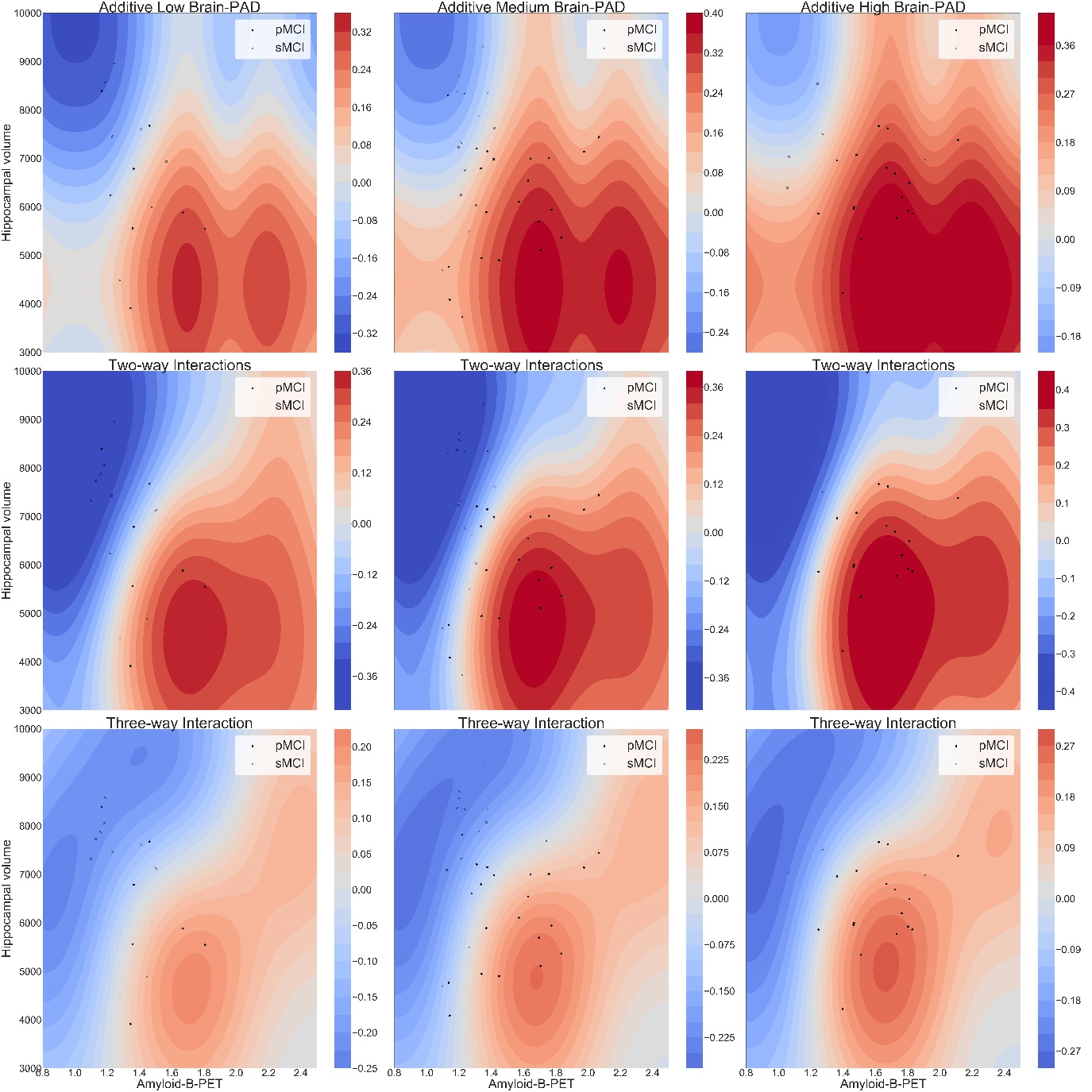
Contour plots show the stable MCI versus progressive MCI decision boundary in biomarker space. First row illustrates the dynamics of the summation of the univariate kernels associated to each biomarker, as Brain-PAD is increased from -4.104 (low Brain-PAD) to 0.575 (medium Brain-PAD) and then finally to 4.411 (high Brain-PAD). Positive values indicate regions where the respective kernel is increasing the probability of a subject to convert to Alzheimer’s disease within three years. Second row depicts the relative contributions of the summation of all two-way interaction kernels. Third row shows the relative contribution of the three-way interaction between all three biomarkers. Comparison of the first row (additive model) and second and third rows (interactive models) conveys the influence of incorporating nonlinear interaction kernels on the biomarker space.

Figure S5. Contour plots for Model 3: Two-way and three-way interactions between P-tau and hippocampal volume, stratified by amyloid-β-PET.


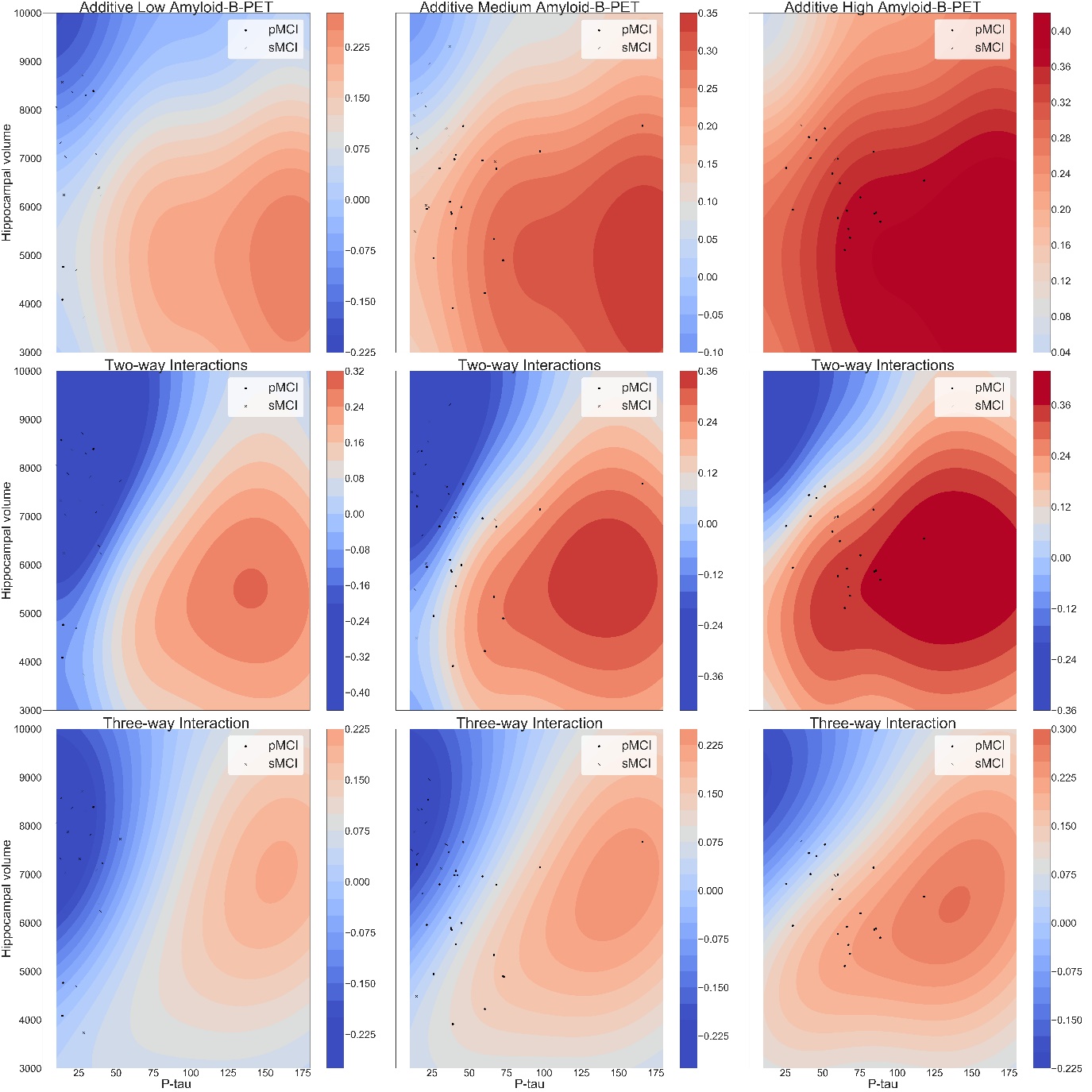


Contour plots show the stable MCI versus progressive MCI decision boundary in biomarker space. First row illustrates the dynamics of the summation of the univariate kernels associated to each biomarker, as Amyloid-β-PET is increased from 1.2 (low Amyloid-β-PET) to 1.6 (medium Amyloid-β-PET) and then finally to 1.9 (high Amyloid-β-PET). Positive values indicate regions where the respective kernel is increasing the probability of a subject to convert to Alzheimer’s disease within three years. Second row depicts the relative contributions of the summation of all two-way interaction kernels. Third row shows the relative contribution of the three-way interaction between all three biomarkers. Comparison of the first row (additive model) and second and third rows (interactive models) conveys the influence of incorporating nonlinear interaction kernels on the biomarker space.

## Tables

Supplementary Table S1. Logistic regression models for classification of stable and progressive MCI

|  | Model | Sensitivity | Specificity | Balanced accuracy | AUC | Bivariate interaction *P* |
| --- | --- | --- | --- | --- | --- | --- |
|  | **Main effects** |  |  |  |  |  |
| 1 | Hippocampal volume, brain-PAD, amyloid-β42 | 0.654 | 0.880 | 0.721 | 0.872 | - |
| 2 | Hippocampal volume, brain-PAD, amyloid-β-PET | 0.635 | 0.872 | 0.700 | 0.851 | - |
| 3 | Hippocampal volume, amyloid-β-PET, P-tau | 0.611 | 0.874 | 0.674 | 0.860 | - |
|  | **Two-way interactions** |  |  |  |  |  |
| 1 | Brain-PAD * amyloid-β42 | 0.652 | 0.872 | 0.700 | 0.851 | 0.211 |
|  | Amyloid-β42 * hippocampal volume | 0.640 | 0.855 | 0.703 | 0.873 | 0.342 |
| 2 | Brain-PAD * amyloid-β-PET | 0.635 | 0.872 | 0.700 | 0.851 | 0.882 |
|  | Hippocampal volume * amyloid-β-PET | 0.610 | 0.870 | 0.673 | 0.854 | 0.321 |
| 3 | Amyloid-β-PET * P-tau | 0.618 | 0.872 | 0.682 | 0.859 | 0.673 |
|  | Amyloid-β-PET * hippocampal volume | 0.619 | 0.879 | 0.684 | 0.861 | 0.712 |
|  | P-tau * hippocampal volume | 0.611 | 0.874 | 0.674 | 0.862 | 0.555 |
| AUC = Area Under the Receiver Operator Characteristic Curve. Brain-PAD = Brain-predicted age difference, P-tau = phosphorylated tau. | | | | | | |

Supplementary Table S2. Relative changes in probability between combinations of dichotomised biomarkers in the NIA-AA classification scheme

| A/T/N classification | Increase probability of progression to AD |
| --- | --- |
| **A-/T-/N-** |  |
| A+ | 10.0 % |
| T+ | 8.0 % |
| N+ | 8.0 % |
| **A+/T-/N-** |  |
| T+ | 9.0 % |
| N+ | 12.5 % |
| **A-/T+/N-** |  |
| A+ | 5.0 % |
| N+ | 2.5 % |
| **A-/T-/N+** |  |
| A+ | 17.5 % |
| T+ | 12.0 % |
| **A+/T+/N+** |  |
| N- | -12.0% |
| T- | -7.0 % |
| A- | -13.0 % |
| Table shows the relative differences in probability of progression to progressive MCI as different dichotomized biomarkers are modified to be either negative or positive in Model 3 using solely the three-way interaction kernel. Results in second column are taken to be the relative difference with respect to the biomarker configuration situated above. | |
